# Involvement of CD36 in Modulating the Decrease of NPY and AgRP Induced by Acute Palmitic Acid Stimulation in N1E-115 Cells

**DOI:** 10.3390/nu9060626

**Published:** 2017-06-17

**Authors:** Yan Ma, Xiaoyi Wang, Hongying Yang, Xu Zhang, Nianhong Yang

**Affiliations:** 1 Department of Nutrition and Food Hygiene, Hubei Key Laboratory of Food Nutrition and Safety, Tongji Medical College, Huazhong University of Science and Technology, Wuhan 430030, China; dezhoumayan@126.com (Y.M.); xiaoyigood1990@163.com (X.W.); zhangxutjmu@icloud.com (X.Z.); 2 Ministry of Education Key Lab of Environment and Health, School of Public Health, Tongji Medical College, Huazhong University of Science and Technology, Wuhan 430030, China; 3 Hubei Provincial Center for Disease Control and Prevention, Wuhan 430030, China; whyanghy2017@sina.com

**Keywords:** palmitic acid, CD36, NPY, AgRP

## Abstract

Central nervous system (CNS) fatty acid sensing plays an important role in the regulation of food intake, and palmitic acid (PA) is the most important long chain fatty acid (LCFA) in the mammalian diet. To explore the effect of PA on central neuropeptide expression and the role of the cluster of the differentiation of 36 (CD36) in the process, N1E-115 cells were cultured with PA in the presence or absence of sulfosuccinimidyl-oleate (SSO), a CD36 inhibitor. Results showed that 10 μmol/L PA significantly reduced NPY and AgRP mRNA expression after 20 min of exposure, while the expression of CD36 was upregulated. The presence of SSO significantly attenuated the decrease of NPY and AgRP expression that was induced by PA alone, although no notable effect on PA- induced CD36 gene expression was observed. In conclusion, our study suggests the involvement of CD36 in the PA-induced decrease of NPY and AgRP in N1E-115 cells.

## 1. Introduction

The central nervous system (CNS) plays an essential role in the regulation of mammalian appetite control and energy expenditure, integrating signals from gastrointestinal afferents and circulating nutrient-related factors to alter behavior and neuroendocrine function [1]. Metabolic sensing neurons have been reported to regulate whole body energy homeostasis in response to ambient levels of glucose and fatty acids (FA) [2,3]. A growing body of evidence suggests that CNS lipid sensing regulates energy balance through insulin secretion and action, adipose deposition, and food intake, etc. [4]. The molecular mechanism involved in this FA sensing by the CNS remains unclear, but it is widely accepted that anorexigenic proopiomelanocortin (POMC)-expressing neurons and orexigenic agouti-related protein (AgRP)/neuropeptide Y (NPY)-expressing neurons are involved in the process [5]. It has been demonstrated that the selective ablation of NPY/AgRP neurons from adult mice causes marked reduction of feeding and body weight [6,7]. Also, the cellular activation of AgRP neurons, which are largely overlapped with NPY neurons in the arcuate nucleus [8], induces acute and robust food intake [9,10]. Research has suggested that NPY/AgRP neurons are the principal inducer of feeding.

High levels of circulating saturated fatty acids are associated with obesity. Previous research has shown that elevated levels of plasma saturated fatty acids, more specifically palmitic acid (PA), cause brain insulin resistance and impair the ability of insulin to activate its intracellular signaling pathways. Furthermore, diets high in PA accelerate obesity [11]. Recent in vitro studies demonstrated that PA treatment also induces both mitochondrial dysfunction and insulin resistance in cultured mouse neuroblastoma Neuro-2a (N2a) cells [12]. Since PA is closely associated with diet- induced obesity, understanding the molecular mechanisms involved in FA sensing by the brain might lead to the identification of novel pharmacological targets for the prevention of diabetes and obesity.

CD36, also known as fatty acid translocase (FAT), is a heavily glycosylated 88-kDa integral membrane protein widely expressed in a variety of tissues and cells [13]. Growing evidence has shown that CD36 is involved in FA responses in brain neurons. Reduction in ventromedial nucleus CD36 expression decreases the expression of both AgRP and POMC, in association with a redistribution of fat from visceral to subcutaneous depots and a marked impairment in insulin sensitivity [14].

In this study, we utilized a mouse neuroblastoma cell line, N1E-115, as a neuronal model system to investigate the role of CD36 in neuronal responses to a normal concentration of PA during acute stimulation.

## 2. Materials and Methods

### 2.1. Cell Culture and Treatment

N1E-115 cells (Bioleaf BIO CO, Shanghai, China) were maintained in Modified Eagle’s medium (MEM, Thermo Fisher, 11095-080, Waltham, MA, USA) containing 4.5 g/L glucose (5.55 mmol/L), 10% fetal bovine serum (FBS, Thermo Fisher, 10099-141, Waltham, MA, USA), 100 units/mL penicillin (Beyotime Institute of Biotechnology, C0222, Haimen, China), and 100 μg/mL streptomycin (Beyotime Institute of Biotechnology, C0222, Haimen, China). Cells were maintained in a humidified incubator under 5% CO_2_ and 95% air at 37 °C.

N1E-115 cells were incubated in MEM with 0.5% (wt/vol) fatty acid-free bovine serum albumin (BSA, EquitechBio, BAH66-0050, Kerrville, TX, USA) in the presence of various concentrations of PA (5, 10, 25, or 50 μmol/L) for different lengths of time (10, 20, 30 min, 1, 2, or 4 h) to investigate the effect of PA on gene expression. A stock solution of 20 mmol/L PA/NaOH was made by dissolving 1282 mg PA (Sigma-Aldrich, 76119, St. Louis, MO, USA) in 250 mL of 0.01 mol/L NaOH at 70 °C for 30 min. Before usage, the final PA concentration of 5, 10, 25, or 50 μmol/L was prepared by adding an appropriate amount of the above stock solution to 330 μL of 30% BSA into 20 mL MEM.

To determine whether CD36 is involved in FA sensing of N1E-115 cells, the cells were pre-incubated with 5 μmol/L CD36 inhibitor SSO (sc-208408, Santa Cruz, CA, USA) for 30 min, then followed by PA treatment for 20 min. A stock solutionof 500 mmol/L SSO was made by dissolving 25 mg SSO in 104 μL dimethyl sulfoxide (DMSO, Sigma-Aldrich, D2650, St. Louis, MO, USA) and the solution was aliquoted and stored at −20 °C. The stock solution was further diluted with DMSO to make 10 mmol/L SSO working solution before use. The concentrationof DMSO in the cell suspension in no case exceeded 0.05%, in order to avoid the effect on cellular fatty acid uptake and metabolism [15].

### 2.2. Cell ViabilityAssay

Cell viability was measured using the Cell Counting Kit-8 (CCK-8, DojinDo, CK04, Kumamoto, Japan) assay, in which cellular dehydrogenase activity in the living cells was detected. Cell viability was expressed as a percentage of the value in the untreated control culture. All experiments were performed in triplicate on three separate occasions.

### 2.3. RNA Extraction and Real-Time Polymerase Chain Reaction (PCR) Analysis

Total RNA was extracted by using TRIZOL (Thermo Fisher, 15596-026, Carlsbad, CA, USA) according to the manufacturer’s manual. A Revert Aid First Strand cDNA synthesis kit (Thermo Fisher, K1622, Carlsbad, CA, USA) was used to synthesize complementary DNA (cDNA) from 3.0 μg of the total RNA. Real-time quantitative PCR was carried out using SYBR Premix Ex TaqTM (Takara Bio, DRR420A, Beijing, China) on an ABI 7900HT real-time PCR system (Applied Biosystems, Forster, CA, USA). The primer sequences used are listed as follows: mouse GAPDH (accession number NM_008084.2): forward primer, 5′-TGCCCAGAACATCATCCCT-3′, and reverse primer, 5′-GGTCCTCAGTGTAGCCCAAG-3′; mouse NPY (accession number NM_023456.3): forward primer, 5′-TAGGTAACAAGCGAATGGG-3′, and reverse primer, 5′-GAGATAGAGCGAGGGTCAG-3′; mouse AgRP (accession number NM_001271806.1): forward primer, 5′-GCTCTGTTCCCAGAGTTCCC-3′, and reverse primer, 5′-CTTGCGGCAGTAGCAAAAGG-3′; mouse POMC (accession number NM_001278581.1): forward primer, 5′-CGGCCCCAGGAACAGCAGCAGT-3′, and reverse primer, 5′-GGGCCCGTCGTCCTTCTCC-3′; and mouse CD36 (accession number NM_001159558.1): forward primer, 5′-TTGAAGGCATTCCCACGTATC-3′, and reverse primer, 5′-CGGACCCGTTGGCAAA-3′. Each sample was performed in triplicate. Gene expression levels were determined by using standard curves generated from a serial diluted reference sample and the housekeeping gene GAPDH was used as an internal control.

### 2.4. Western Blot Analysis

Proteins were resolved by SDS-PAGE through either 4–15% gradient or 15% polyacrylamide gels, then transferred to polyvinylidene difluoride membraneand blocked for 1.5 h with 5% milk in tris buffered saline (TBS)-0.05% Tween. The membranes were then incubated with the primary antibody CD36 (1:100 dilution, sc-9154, Santa Cruz, CA, USA), or GAPDH (1:1000 dilution, #2118, Cell Signaling, Danvers, MA, USA), overnight at 4 °C after blocking. After being washed with TBS-T three times, the membranes were next incubated for 1 h with horseradish peroxidase-conjugated secondary antibodies (1:3000 dilution, #7074, Cell Signaling, Danvers, MA, USA). Immunoreactive bands were detected using a commercial chemiluminescence enhancement kit (Super Signal West Pico, Pierce, NE, USA) according to the manufacturer’s instructions. Quantitative analysiswas performed by Quantity One 4.62 software (Bio-Rad, Hercules, CA, USA).

### 2.5. Statistical Analysis

All data were expressed as mean ± SEM and differences were considered statistically significant at *p* < 0.05. Statistical analyses were conducted using one-way ANOVA analysis of variance followed by Tukey’s post hoc test with SPSS 16.0 software package (SPSS Inc., Chicago, IL, USA).

## 3. Results

### 3.1. PA Treatment Does Not Affect N1E-115 Cell Viability

A CCK-8 assay was performed to evaluate the effect of PA treatmenton cell viability. None of the tested concentrations or times showed any significant effects on the viability of the cultured N1E-115 cells (F value = 0.232, *p* > 0.05).

### 3.2. PA Treatment Affects NPY and AgRP but Not POMC Expression in N1E-115 Cells

**Figure 1:**
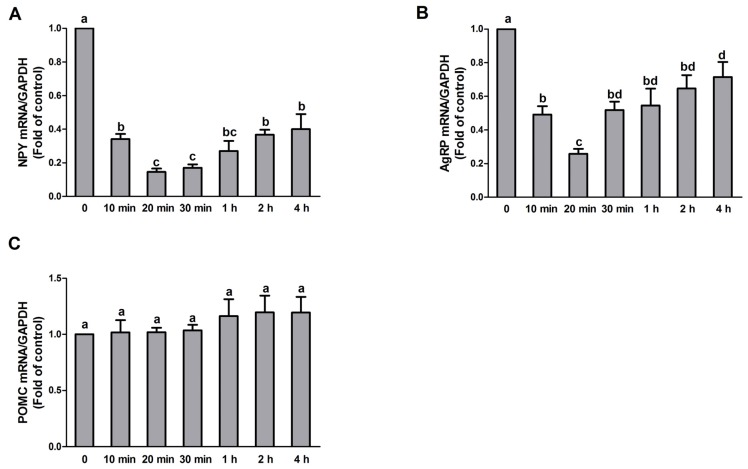
Time-dependent effect of PA on NPY, AgRP, and POMC expression in N1E-115 cells. Quantitative real-time PCR analysis of NPY, AgRP, and POMC performed with RNA extracted from N1E-115 cells treated with 10 μmol/L PA for different durations (**A**–**C**), respectively. Quantified mRNA levels were normalized to GAPDH and presented relative to 10 μmol/L PA for 0 min. Data are expressed as means ± SEM of three different experiments. Groups sharing different letters above the bars indicate statistically significant differences (*p* < 0.05), while those denoted by the same letters are insignificant (**A**–**C**). PA: palmitic acid group.

To determine the effect of PA treatment on NPY, AgRP, and POMC gene expression, N1E-115 cells were treated with 10 μmol/L PA for 0, 10, 20, 30 min, 1, 2, and 4 h, respectively. Quantitative real-time PCR results showed that the expression of both NPY and AgRP were decreased upon PA exposure and the lowest levels were observed at the 20 min timepoint (NPY: F value = 41.745, *p* < 0.05; AgRP: F value = 13.857, *p* < 0.05) (Figure 1A,B). There was no significant change in POMC expression following PA exposure (F value = 0.117, *p* > 0.05) (Figure 1C).

**Figure 2:**
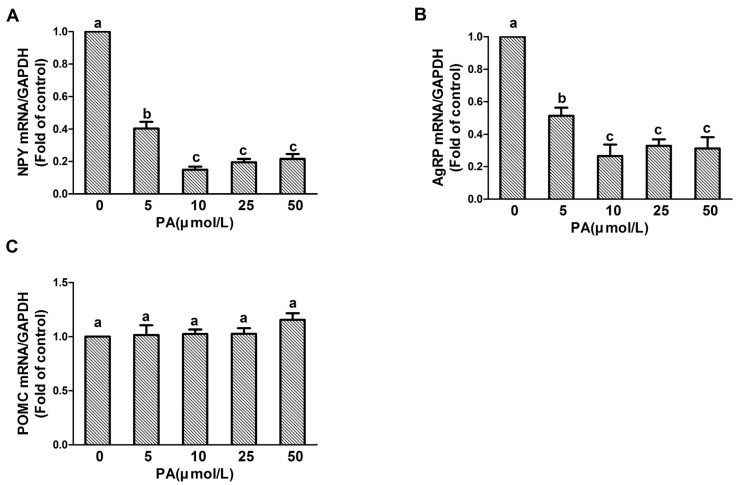
Dose-dependent effect of PA on NPY, AgRP, and POMC expression in N1E-115 cells. Quantitative real-time PCR amplification of NPY, AgRP, and POMC performed with RNA extracted from N1E-115 cells following treatment with differing concentrations of PA for 20 min (**A**–**C**), respectively. Quantified mRNA levels were normalized to GAPDH and presented relative to 0 μmol/L PA for 20 min. Data are expressed as means ± SEM of three different experiments. Groups sharing different letters above the bars indicate statistically significant differences (*p* < 0.05), while those denoted by the same letters are insignificant (**A**–**C**). PA: palmitic acid group.

Since 20 min of PA treatment showed the most significant effects on NPY and AgRP genes, we decided that 20 min was to be used as the appropriate timepoint for subsequent analyses. The cells were then treated with different doses of PA for 20 min and the expressions of NPY, AgRP, and POMC were determined. As shown in Figure 2A,B, all tested dosages of PA significantly reduced NPY and AgRP gene expression, and the 10 μmol/L treatment displayed the most significant reduction (NPY: F value = 90.033, *p* < 0.05; AgRP: F value = 16.041, *p* < 0.05). As expected, different dosages of PA did not alter POMC gene expression in the cells (F value = 0.542, *p* > 0.05) (Figure 2C).

### 3.3. CD36 Is Involved in the PA-Dependent Regulation of NPY and AgRP Gene Expression

**Figure 3:**
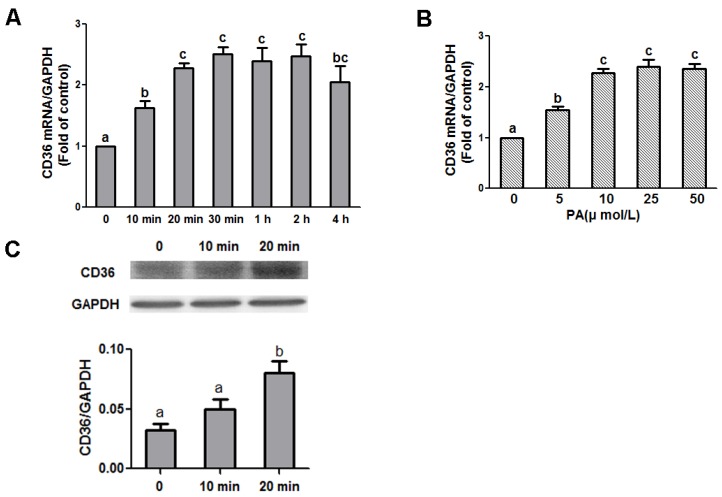
Effect of PA on CD36 expression in N1E-115 cells. Quantitative real-time PCR amplification of CD36 performed with RNA extracted from N1E-115 cells treated with 10 μmol/L PA at different time points (**A**) or different concentrations of PA for 20 min (**B**), respectively. (**C**) Representative Western blot analysis of CD36 protein in N1E-115 cells induced by 10 μmol/L PA at 0, 10, and 20 min. Data are expressed as means ± SEM of three different experiments. Groups sharing different letters above the bars indicate statistically significant differences (*p* < 0.05), while those denoted by the same letters are insignificant (**A**–**C**). PA: palmitic acid group.

The role CD36 plays in the effect of PA on NPY and AgRP was examined. N1E-115 cells were treated with varying concentrations of PA for 20 min or with 10 μmol/L PA for different time periods. As shown in Figure 3A, 10 min after the cells were treated with 10 μmol/L PA, the CD36 mRNA level nearly doubled, and then continued to increase until it reached the maximum level at 20 min, which was maintained for the duration of the experiment (F value = 7.192, *p* < 0.05). Similar to what we observed for NPY and AgRP expression, the 10 μmol/L dose displayed the strongest effect on CD36 expression (F value = 11.107, *p* < 0.05) (Figure 3B). The CD36 protein level was increased when the cell was treated with 10 μmol/L PA for 20 min (F value = 45.464, *p* < 0.05) (Figure 3C).

**Figure 4:**
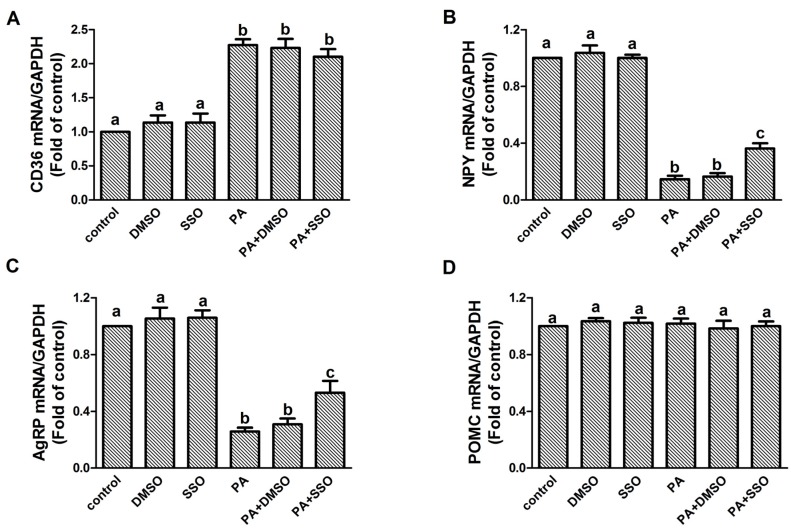
Effect of CD36 blockade on the regulation of NPY and AgRP by PA in N1E-115 cells. N1E-115 cells were pre-incubated with 5 μmol/L CD36 inhibitor SSO for 30 min before being treated with 10 μmol/L PA for 20 min. The mRNA expression levels of CD36 (**A**); NPY (**B**); AgRP (**C**); and POMC (**D**) were analyzed by real-time PCR. Quantified mRNA levels were normalized to GAPDH and expressed as fold changes relative to the vehicle-treated control. Data are expressed as means ± SEM of three different experiments. Groups sharing different letters above the bars indicate statistically significant differences (*p* < 0.05), while those denoted by the same letters are insignificant (**A**–**D**). DMSO: DMSO only group; SSO: 5 μmol/L of SSO group; PA: 10 μmol/L palmitic acid group; PA+DMSO: N1E-115 cells were treated with 10 μmol/L palmitic acid for 20 min after pretreatment with DMSO for 30 min; PA+SSO: N1E-115 cells were treated with 10 μmol/L palmitic acid for 20 min after pretreatment with 5 μmol/L of SSO for 30 min.

We next tested whether the inhibition of CD36 affects the ability of PA to induce a reduction in NPY and AgRP expression by including the CD36 inhibitor SSO in the culture medium. SSO is achemical that binds irreversibly to CD36 and inhibits fatty acid uptake [16]. Our results showed that SSO pretreatment of cells did not significantly affect the increase of CD36 expression induced by PA (F value = 10.643, *p* < 0.05) (Figure 4A). However, the PA-induced decrease of NPY and AgRP was significantly attenuated by the pretreatment of 5 μmol/L SSO (NPY: F value = 82.667, *p* < 0.05; AgRP: F value = 25.054, *p* < 0.05) (Figure 4B,C).

## 4. Discussion and Conclusions

It has been documented that CNS nuclei respond to energy status changes, alter the expression of specific neurotransmitters/neuromodulators, and result in changes in energy intake and expenditure [1,17]. Recent evidence has indicated the important regulatory roles of fatty acids in CNS energy regulation [18]. In the wake of the obesity pandemic, understanding how peripheral metabolites regulate energy homeostasis is of great importance. Since peripheral nutrient sensing and its dysregulation represent key components of diet-induced obesity, impaired FA sensing in the CNS may also be involved in the development of obesity [4]. Indeed, saturated fatty acids, such as palmitate, which is also increased in the CNS upon high-fat consumption [11], acutely cause leptin resistance in the CNS [19]. CD36 is known to be an important membrane protein that mediates LCFA transport [20] and is involved in CNS FA sensing [21]. The expression level of CD36 and its ability to transport LCFA affects the intracellular long chain fatty acyl–CoAs (LCFA-CoAs) concentrations. We have noted that a 16-h fast followed by a 2-h high-fat diet refeeding resulted in a decrease of NPY/AgRP expression and an increase in CD36 expression in rats resistant to diet-induced obesity, but not in rats susceptible to diet-induced obesity (data unpublished), suggesting that brain CD36 may be associated with neuropeptide expression. However, research studying the direct relation between CD36 and appetite neuropeptides is sparse. In this study, we investigated the role of CD36 on PA-induced neuropeptide changes in N1E-115 cells, and demonstrated that the blockade of the CD36 pathway attenuates the inhibition of NPY/AgRP by PA.

PA is the most common saturated fatty acid in human diets; it accounts for almost 65% of the saturated fatty acids and 32% of the total fatty acids in human serum [22]. In fasted anesthetized dog cerebral spinal fluid, free fatty acid levels were estimated at 25 μmol/L [23]. In this study, we attempted to mimic the physiological process of refeeding by stimulating the cells with PA for a short period of time. N1E-115 cells, a murine neuroblastoma cell line, which is commonly used to study the changes of neurotransmitters and their receptors [24,25], were tested. Consistent with previous reports that the central administration of oleic acid decreased the hypothalamic NPY mRNA levels in rodents [26,27], our study demonstrated that PA reduced NPY and AgRP expression in a dose- and time- dependent manner. A CCK-8 assay confirmed that the changes in gene expression levels were not due to varied cell viabilities. Even though Le Foll et al. noted that in vivo CD36 depletion caused a comparable decrease in AgRP and POMC expression in the ventromedial nucleus of the hypothalamus in rats [14], we noted that POMC gene expression was not altered by the treatment of PA, which is consistent with previous reports showing differential responses in the expression of different neuropeptides upon different stimuli [26,28], suggesting that NPY/AgRP neurons may be more highly sensitive to metabolite changes than POMC.

Using a CD36-specific inhibitor, SSO, we further revealed that CD36 is involved in the regulation of NPY and AgRP genes by PA in N1E-115 cells. Animal experiments showed that ablation of the ventromedial hypothalamic CD36 gene in obesity-prone rats significantly increased both the body weight and body fat of the animals [29]. It is known that several free fatty acid responsive transcription factors, including peroxisome proliferator-activated receptor (PPAR) D and PPARA, can transcriptionally upregulate CD36 expression. Consistent with this finding, we also noted that PA treatment upregulates CD36 expression in NIE-115 cells, which suggests the existence of a positive feedback pathway in CD36-mediated FA sensing [30]. Notably, we also found that the expression of CD36 was gradually increased in N1E-115 cells along with the decrease of NPY and AgRP with the addition of normal physiological concentrations of PA, while the inhibition of the function of CD36 significantly attenuated the decrease of NPY and AgRP that was induced by PA. CD36 is a multi-functional protein and exhibits a variety of different signaling functions upon binding with different ligands [31]. SSO specifically binds to CD36 lysine 164, resulting in an inhibition of the FA transport function of this protein [32], but could also inhibit CD36-dependent FA signaling [31,33]. It is known that cellular accumulation of CNS LCFA-CoAs decreases NPY/AgRP expression and food intake [34], while the expression level of CD36 and its ability to transport LCFA affects the intracellular LCFA-CoAs concentrations. In the current study, the decrease of NPY and AgRP induced by PA were not completely reversed by SSO, which indicated that there might exist other factors affecting the expression of NPY and AgRP. Besides CD36, other transporters such as Glut2 have also been proposed to be involved in FA sensing. It has been reported that disruption of the Glut2 gene caused decreased food intake [35,36,37] and that free FA affected the expression of Glut2 [38]. In addition, CNS FA sensing is also controlled by peripheral hormones, such as insulin, leptin, and ghrelin [39,40,41]. Therefore, further study is warranted to determine whether other transporters or factors are involved in the regulation of AgRP and NPY expression in response to PA.

In conclusion, our study demonstrated that PA reduces NPY and AgRP gene expression without affecting POMC expression in N1E-115 cells, and that plasma membrane CD36 is involved in the regulation of NPY and AgRP genes by PA, which remains to be proved in other cell lines and in vivo models. Further studies to explore the underlying mechanism of these patterns are warranted.
